# LAMB3 mediates metastatic tumor behavior in papillary thyroid cancer by regulating c-MET/Akt signals

**DOI:** 10.1038/s41598-018-21216-0

**Published:** 2018-02-09

**Authors:** Seung-Nam Jung, Hyun Sil Lim, Lihua Liu, Jae Won Chang, Young Chang Lim, Ki Sang Rha, Bon Seok Koo

**Affiliations:** 1 Department of Otolaryngology-Head and Neck Surgery, Research Institute for Medical Science, Chungnam National University College of Medicine, Daejeon, Republic of Korea; 2 Department of Medical Science, College of Medicine, Chungnam National University, Daejeon, Republic of Korea; 3 Department of Otolaryngology-Head and Neck Surgery, Research Institute for Medical Science, Konkuk University College of Medicine, Seoul, Republic of Korea

## Abstract

Laminin subunit beta-3 (LAMB3) encodes one of the three subunits of LM-332, a protein of the extracellular matrix secreted by cultured human keratinocytes. While LAMB3 is involved in the invasive and metastatic abilities of several tumor types, including those found in the colon, pancreas, lung, cervix, stomach, and prostate, its mechanism of action in thyroid cancer has not been investigated previously. Our results show that LAMB3 is up-regulated in papillary thyroid cancer, and that its suppression reduces cell migration/invasion via down-regulation of epithelial‒mesenchymal transition-associated proteins (N-cadherin, vimentin, slug) and inhibition of matrix metalloproteinase 9. LAMB3 suppression also significantly decreases Akt phosphorylation and inhibits the transcription of c-MET, reducing its activation. These results suggest that LAMB3 leads to tumor invasion via Akt activation induced by the HGF/c-MET axis in papillary thyroid cancer cells. Our findings reveal a novel mechanism of action for LAMB3 in papillary thyroid cancer cells.

## Introduction

Thyroid cancer is the most common type of endocrine malignancy, and its incidence has grown rapidly over the last few decades^1,2^. Papillary thyroid cancer (PTC) is the major type, accounting for 80‒85% of all thyroid malignancies^3^. Most PTC patients have a good prognosis, and the disease has a low mortality rate. However, PTC frequently metastasizes to lymph nodes, and nodal metastasis can increase both the locoregional recurrence and cancer-specific mortality rates^4–6^. In addition, certain cases exhibit aggressive clinical characteristics, including invasion and metastasis^7^. Therefore, it is essential to identify potential molecular biomarkers associated with the aggressiveness of PTC that may be beneficial for improving treatment.

PTCs usually possess BRAF mutations or rearranged in translation (RET)/PTC rearrangements. Many of the genetic changes, including RET/PTC gene rearrangements and RAS and BRAF mutations, cause activation of the mitogen-activated protein kinase (MAPK) and phosphatidylinositol-3-kinase (PI3K)/Akt signaling pathways^8^. The PI3K/Akt pathway has a fundamental role in thyroid tumorigenesis and represents an attractive target for pharmaceutical development in a variety of malignancies^9,10^.

Receptor tyrosine kinases (RTKs) are a family of cell surface receptors for growth factors, hormones, cytokines, neurotrophic factors, and other extracellular signaling molecules. RTKs mediate key signaling pathways that are involved in cell proliferation, differentiation, survival, and cell migration^11^. The RTK c-Met is the cell surface receptor for hepatocyte growth factor (HGF), also known as scatter factor. Activation of the HGF/c-Met axis prevents apoptosis via activation of PI3K and subsequent Akt activation^12–14^. In a previous study, we found that the HGF/c-Met pathway activation is associated with subclinical lymph node metastasis of the central neck in PTC^15^.

The laminins are important and biologically active components of the basal lamina, influencing cell differentiation, migration, and adhesion, as well as cell proliferation and survival. Laminin subunit beta-3 (LAMB3) encodes one of the three subunits of LM-332, a protein of the extracellular matrix secreted by cultured human keratinocytes. The α3, β3, and γ2 chains of LM-332 are encoded by three distinct genes, LAMA3, LAMB3, and LAMC2, respectively. LAMB3 is integral to the invasive and metastatic abilities of several tumor types found in the colon, pancreas, lung, cervix, and prostate^16–18^. However, the molecular role of LAMB3 in thyroid cancer has not yet been fully elucidated. We observed LAMB3 up-regulation in PTC cells compared with cells from normal thyroid tissue. Therefore, we hypothesized that LAMB3 overexpression is a common finding in thyroid cancer and might be important for the aggressive features, such as metastasis, of PTC. We investigated the functional significance of LAMB3 in PTC and identified a novel molecular mechanism.

## Materials and Methods

### Cell lines and materials

The normal human thyroid cell line N-thy-ori and the human thyroid cancer cell lines B-CPAP, K1, and TPC-1 were obtained from the Korean Cell Line Bank (Seoul, South Korea). B-CPAP, K1, and TPC-1 cells were maintained in high-glucose DMEM (Gibco, Grand Island, NY, USA). N-thy-ori was maintained in RPMI 1640 (Gibco). All cells were supplemented with 10% fetal bovine serum (FBS) and 100 u/mL penicillin‒streptomycin (Gibco) and grown at 37 °C with 5% CO_2_ under humidified conditions. LY294002, an Akt inhibitor, was purchased from Cell Signaling Technology Inc. (#9901; Danvers, MA, USA).

### RNA isolation and reverse transcription-polymerase chain reaction (RT-PCR)

For RT-PCR analysis, total cellular RNA was extracted using Trizol reagent (Invitrogen, Carlsbad, CA, USA), reverse transcribed, and amplified using specific primers for LAMB3, matrix metalloproteinases (MMP) 2 and MMP9, and glyceraldehyde 3-phosphate dehydrogenase (GAPDH), as described previously^19^. The primer sequences were as follows: LAMB3-F: 5′-CCA AGC CTG AGA CCT ACT GC-3′/LAMB3-R: AAG CTG GAA TCT CCT GTC CA-3′, MMP2-F: 5′-ATG ACA GCT GCA CCA CTG AG-3′/MMP2-R:F: 5′-ATT TGT TGC CCA GGA AAG TG-3′, MMP9-F: 5′-TTG ACA GCG ACA AGA AGT GG-3′/MMP9-R:F: 5′-GCC ATT CAC GTC GTC CTT AT-3′ and GAPDH-F: 5′-ACC CAG AAG ACT GTG GAT GG-3′/GAPDH-R:F: 5′-TTC TAG ACG GCA GGT CAG GT-3′. PCR products were separated by electrophoresis on 2% agarose gels containing ethidium bromide.

### Western blot analysis

Cells were lysed in buffer containing 150 mM NaCl, 1.0% nonidet-P40, 0.5% sodium deoxycholate, 0.1% sodium dodecyl sulfate (SDS), 50 mM Tris, pH 8.0, and a protease inhibitor cocktail (Roche Applied Science, Vienna, Austria, pH 7.4). Frozen tissue samples stored in liquid nitrogen were cut into pieces with scissors. Each sample was homogenized in lysis buffer at a ratio of 1:20 w/v. After a centrifugation at 13,000 rpm for 20 min step the supernatant was used to measure the total protein. Electrophoresis was performed as described previously^20^. The following primary antibodies were used for western blot analysis: anti-LAMB3 (1:1000; OriGene Technologies Inc. Rockville, MD, USA) anti-phospho-Akt (Ser 473), anti-total Akt, anti-vimentin, anti-Slug, anti-Snail, anti-β-actin (1:1,000; Cell Signaling Technology Inc, Danvers, MA, USA), LAMA3, LAMC2, anti-E-cadherin, and anti-N-cadherin (1:1,000; Santa Cruz Biotechnology, Santa Cruz, CA, USA). Following incubation with the corresponding horseradish peroxidase-conjugated secondary antibodies (1:10,000; Santa Cruz Biotechnology), immunoreactive bands were visualized by enhanced chemilumi-nescence (ECL) detection.

### Transient transfection

Transient transfection was performed once cells reached 60% confluence using Lipofectamine RNAi max reagent (Invitrogen) following the manufacturer’s standard protocol. LAMB3 siRNA (sense: 5′-GUG UGU GCA AGG AGC AUG U(dTdT)-3′; antisense: 5′-ACA UGC UCC UUG CAC ACA C(dTdT)-3′) and LAMB3 siRNA^#^ (sense: 5′-UCA UCU GCC GCC UUU GCU U(dTdT)-3′; antisense: 5′-AAG CAA AGG CGG CAG AUG A(dTdT)-3′), or negative control siRNAs (#SN-1003, bioneer) were acquired from Bioneer (Daejeon, Korea).

### Cell proliferation assay

Cell were seeded at 5 × 10^3^/well in 96-well plates in DMEM containing 10% FBS. After transfection with siRNA for 48 h, TPC-1 and B-CPAP cell viabilities were measured using the Cell Proliferation Reagent WST-1 (Roche Diagnostics Corporation, Indianapolis, IN, USA). WST-1 formazan was quantitatively measured at 450 nm using an enzyme-linked immunosorbent assay reader. Results are presented as percentages, relative to control cells.

### Cell migration and invasion assay

Transwell membranes (24-well, Costar, Cambridge, MA, USA) were either coated with Matrigel for 6 h for invasion assays or were used without Matrigel for migration assays. Cells (0.5 × 10^5^) in serum-free medium were seeded into each upper chamber, and 600 μl medium supplemented with 10% FBS were added to each lower chamber. After incubation for 24 h, cells adhering to the upper surface of the membrane were removed with a cotton swab. Cells that had invaded or migrated, which were adhered to the lower surface, were stained with 0.1% crystal violet and counted in four representative fields by light microscopy (200 × magnification).

### Zymography

TPC-1 cells (1 × 10^5^/well) cultured in 6-well plates were transfected with LAMB3 siRNA or negative control siRNA for 48 h. MMP activity was then measured in the conditioned culture media by substrate gel electrophoresis using 8% SDS-PAGE gels containing 0.2% gelatin. Conditioned medium samples were adjusted to equal protein concentrations, mixed with sample buffer (250 mM Tris-HCl, pH 6.8, 40% glycerol, 8% SDS, 0.01% bromophenol blue), and loaded onto gels for protein separation by electrophoresis. To remove the SDS, the gels were soaked three times for 30 min at room temperature in Triton buffer (2.5% Triton X-100 in PBS). Gels were then incubated in Zymogram development buffer (#161–0766, Bio-Rad, Hercules, CA, USA) for 24 h at 37 °C and stained with EZblue reagent (Sigma-Aldrich, Louis, MO, USA, #G1041). Gels were destained to obtain clear bands, and quantitative results were obtained by densitometry.

### Screening for RTK expression in cells

RTKs were examined using a western blot array (Proteome Profiler Human Phospho-RTK Array Kit, ARY001B, R&D Systems, Minneapolis, MN, USA) according to the manufacturer’s instructions. All experiments were performed in duplicate. In brief, 1 × 10^7^ cells/ml were washed in PBS and extracted using the appropriate kit buffer supplemented with protease inhibitor cocktail (Sigma-Aldrich). Extracts were centrifuged, and the supernatants were diluted with sample buffer and applied to nitrocellulose membranes previously spotted with 49 anti-kinase receptor antibodies. RTKs were then detected using an HRP-conjugated anti-phospho-tyrosine antibody and chemiluminescence.

### Statistical analysis

All *in vitro* experiments were repeated three times, and statistical significance was analyzed using two-sided Student’s t-test. Data are presented as means ± standard deviation (SD), and a P value < 0.05 was considered statistically significant (*P < 0.05).

## Results

### LAMB3 expression in thyroid cancer patient tissues and cell lines

**Figure 1:**
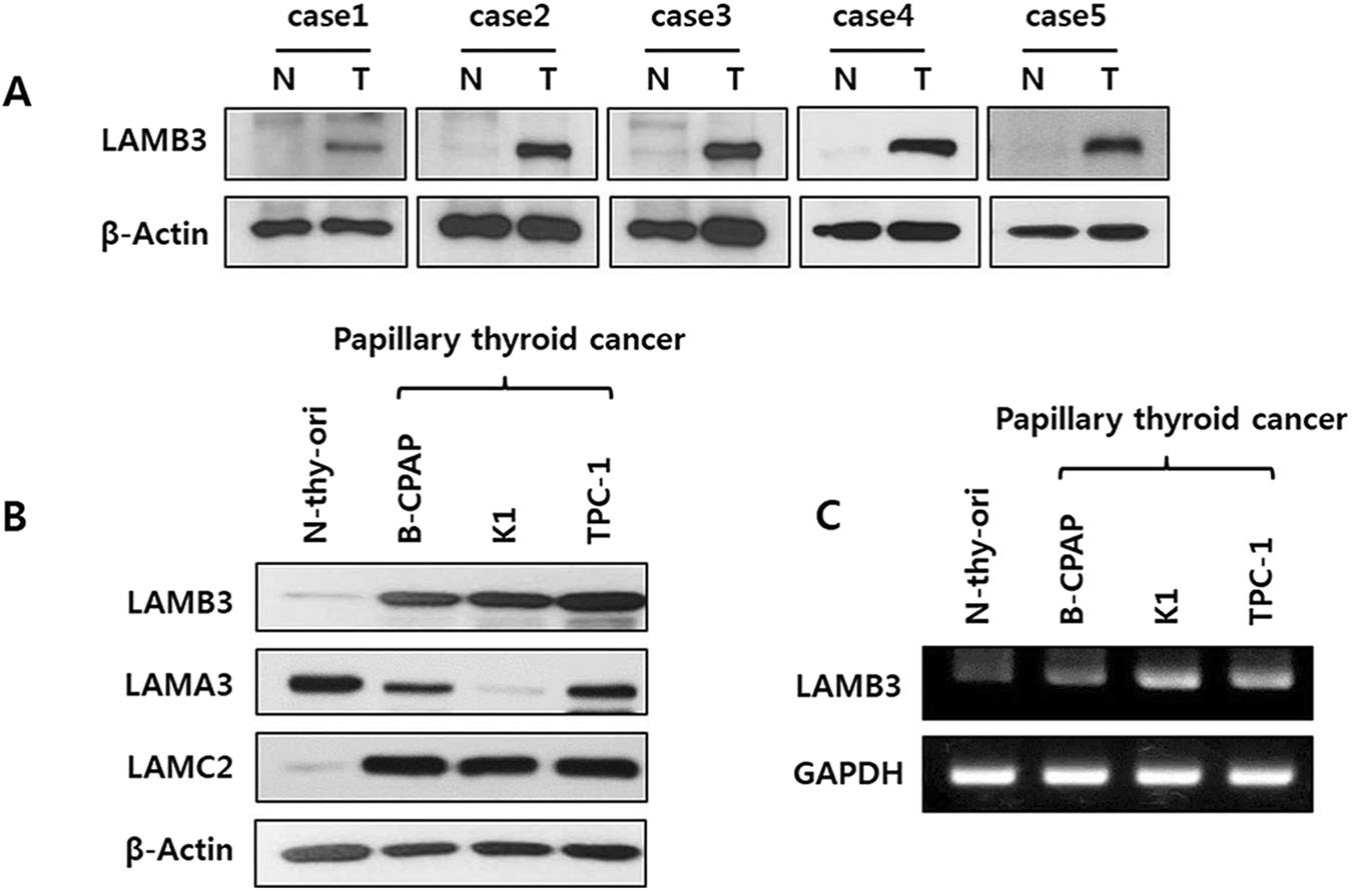
LAMB3 expression in thyroid cancer cells. (**A**). Tissue samples obtained from thyroid cancer patients were examined by western blot analysis using an anti-LAMB3 antibody. (**B**). Cell lysates were prepared from a normal thyroid cell line (N-thy-ori) and from three thyroid cancer cell lines (B-CPAP, K1, and TPC-1) and examined by western blot analysis. (**C**). Cell lysates were subjected to RT-PCR analysis. Representative images of three independent experiments are shown.

We first evaluated LAMB3 expression in normal and tumor tissues derived from the same thyroid cancer patients. Considerably higher expression of LAMB3 protein levels was detected in all five tumor samples relative to the normal tissues (Fig. 1A). The expression levels of LAMB3 mRNA and protein were also examined in a normal thyroid cell line (N-thy-ori) and in three PTC cell lines (B-CPAP, K1, and TPC-1). All PTC cell lines demonstrated notably higher LAMB3 expression at both the mRNA and proteins levels (Fig. 1B,C). The results indicate that overexpression of LAMB3 is correlated with tumorigenesis in PTC. We also examined the expression of LAMA3 and LAMC2 in PTC cell lines because many studies revealed that these could play an important role in carcinogenesis of several cancers (Fig. 1B). LAMC2 protein levels were increased in PTC cell lines compared to normal thyroid cell line (N-thy-ori). However, in our study we just focused on the regulation and functions of LAMB3 in PTCs.

### LAMB3 promotes cell migration and invasion, but not proliferation, in PTC cells

**Figure 2:**
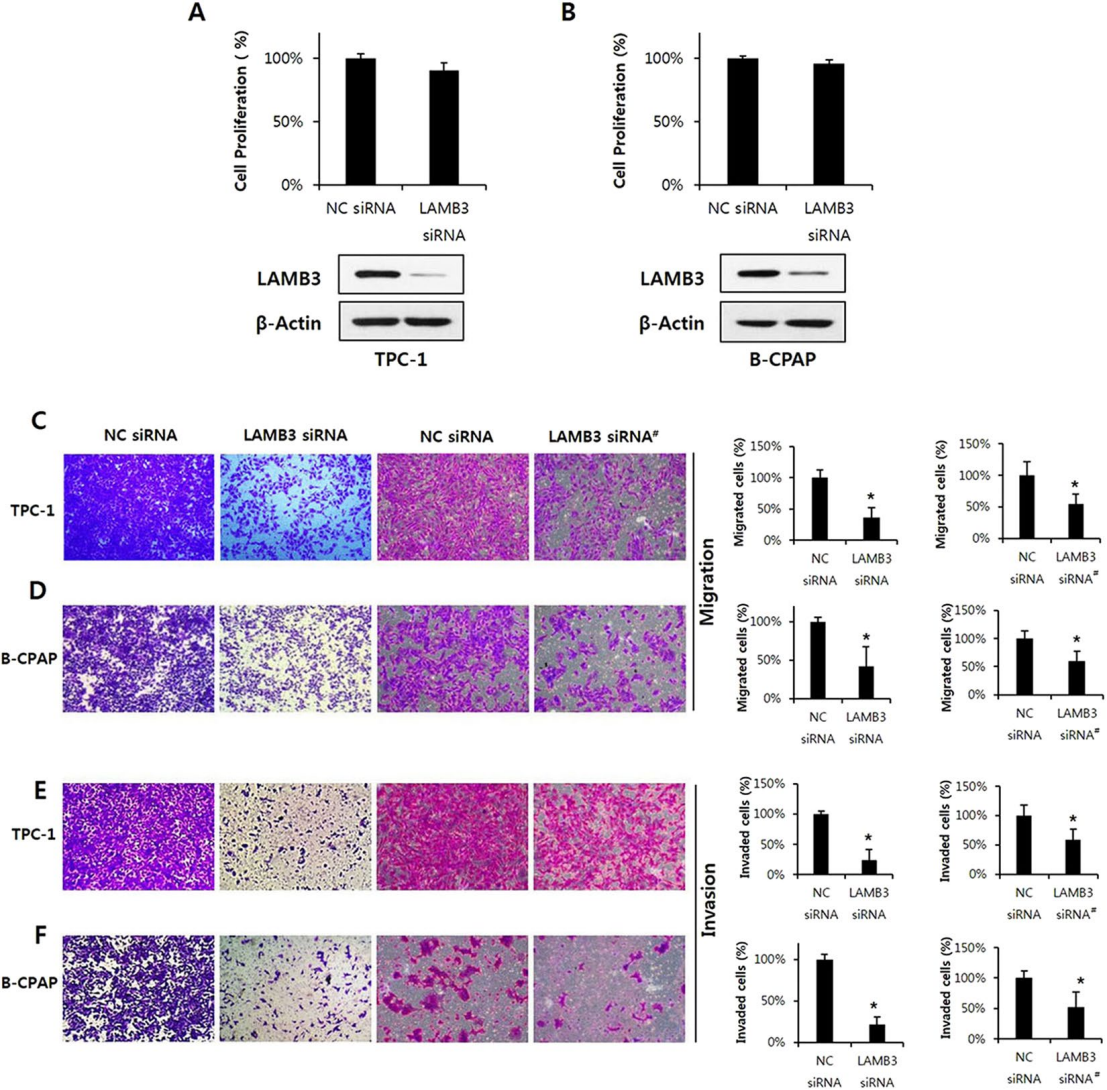
LAMB3 promotes migration and invasion but does not alter proliferation of papillary thyroid cancer cells. Proliferation of TPC-1 (**A**) and B-CPAP (**B**) cells was analyzed by WST-1 assay after transfection with LAMB3 siRNA or negative control siRNA for 48 h. LAMB3 protein levels after transfection were examined by western blot analysis. TPC-1 (**C**,**E**) and B-CPAP cells (**D**,**F**) were transiently transfected with LAMB3 siRNAs or negative control siRNA for 48 h. Cells were allowed to migrate for 24 h in Transwell chambers (Cell Migration) or for 48 h in chambers coated with Matrigel (Cell Invasion). Each figure is representative of three independent experiments. LAMB3 siRNA^#^, second LAMB3 siRNA; *P < 0.05,.

We hypothesized that LAMB3 participates in PTC tumorigenesis and metastasis. To investigate the functional significance and mechanism of action of LAMB3 in PTC, we used two PTC cell lines, TPC-1 (carrying RET/PTC-1) and B-CPAP (carrying BRaf^V600E^). We first examined the effect of LAMB3 on cell proliferation. TPC-1 and B-CPAP cells were transiently transfected with LAMB3 siRNA or a negative control siRNA for 48 h, and cell proliferation was detected by WST-1 assay. LAMB3 knockdown specifically down-regulated LAMB3 but had no significant effect on cell proliferation in the two PTC lines (Fig. 2A,B). These results suggest that LAMB3 is not associated with cell proliferation in PTC cells. Cell migration and invasion have been recognized as key steps in tumor metastasis. To determine the effects of LAMB3 on cell migration and invasion, TPC-1 and B-CPAP cells were transiently transfected with LAMB3 siRNAs or with negative control siRNA for 48 h. PTC cells were then allowed to migrate for 24 h in Transwell chambers (cell migration) or for 48 h in chambers with Matrigel (cell invasion). Although it had no effect on cell proliferation, LAMB3 knockdown considerably suppressed the migration and invasion of TPC-1 and B-CPAP cells (Fig. 2C‒F). These results clearly show that LAMB3 promotes the migration and invasion of PTC cells.

### LAMB3 regulates epithelial‒mesenchymal transition (EMT)-related proteins and metastasis-related proteins

**Figure 3:**
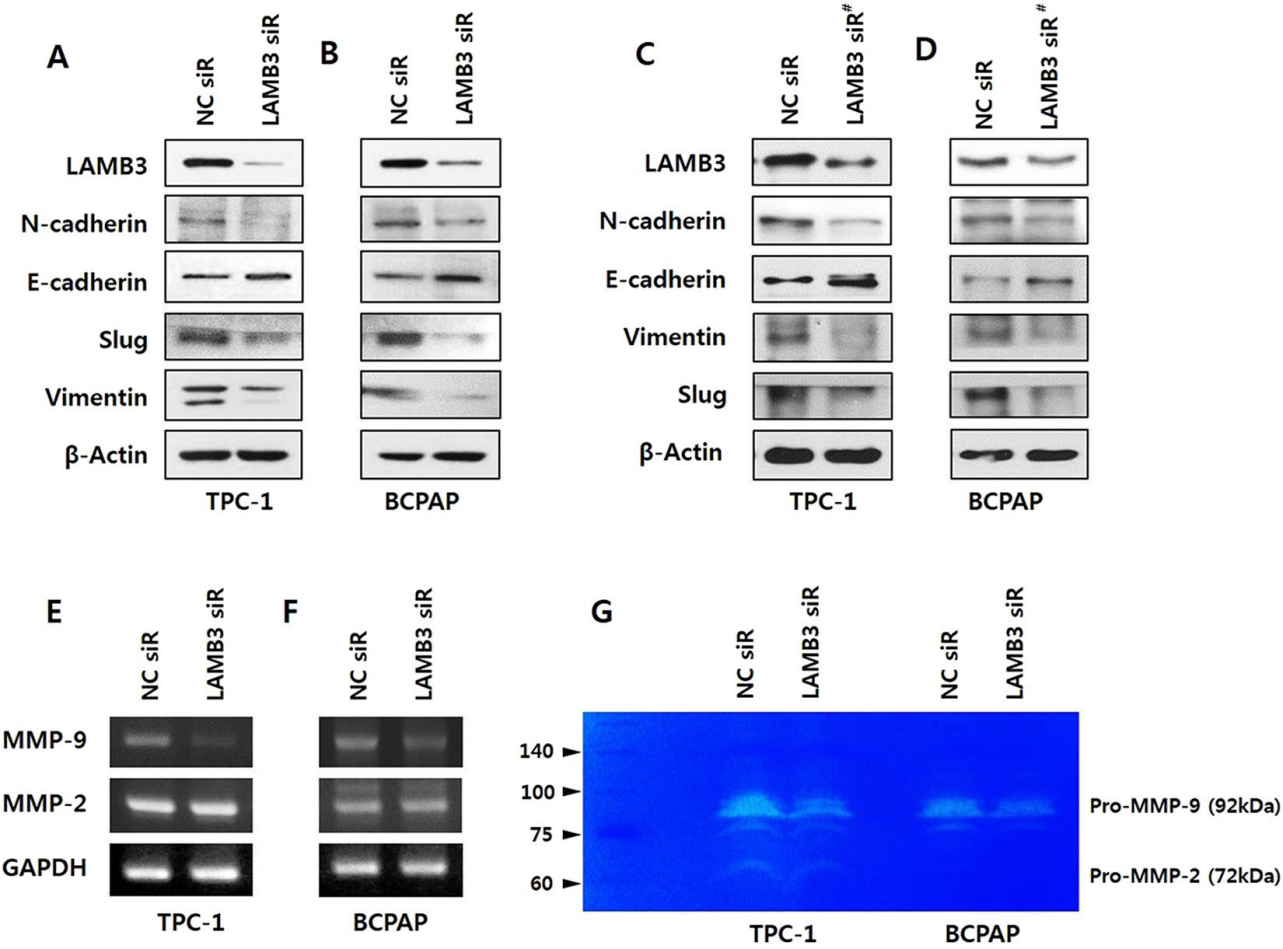
LAMB3 regulates EMT-related proteins and metastasis-related proteins. TPC-1 (**A**,**C**) and B-CPAP (**B**,**D**) cells were transiently transfected with LAMB3 siRNAs or negative control siRNA for 48 h. After transfection, the levels of the EMT-related proteins N-cadherin, E-cadherin, Slug, and vimentin were evaluated by western blot analysis. MMP2 and MMP9 mRNA levels in TPC-1 (**E**) and B-CPAP (**F**) cells were analyzed by RT-PCR after transfection with LAMB3 siRNA or negative control siRNA. GAPDH was used as an internal control. (**G**). Using gelatin zymography, pro-MMP2 and pro-MMP9 levels were measured in TPC-1 and B-CPAP cells after transfection with LAMB3 siRNA or negative control siRNA. Each figure is representative of three independent experiments. LAMB3 siRNA#, second LAMB3 siRNA.

The association between EMT and cell invasion has been demonstrated in cancer progression. To investigate whether LAMB3 promotes EMT, we examined both epithelial and mesenchymal markers, E-cadherin, N-cadherin, and vimentin, as well as the well-known EMT marker Slug, by immunoblot analysis. LAMB3 knockdown caused a significant decrease in N-cadherin, vimentin and Slug levels and increase in E-cadherin in TPC-1 and B-CPAP cells (Fig. 3A–D). Matrix MMPs, particularly MMP2 and MMP9, are integral to the processes of invasion and metastasis. To determine whether LAMB3 regulates MMPs, we investigated the expression and secretion of MMP2 and MMP9 in both PTC cell lines. Pro-MMP9 mRNA expression was significantly reduced by LAMB3 knockdown in TPC-1 and B-CPAP cells (Fig. 3E,F) and Pro-MMP9 secretion, analyzed by gelatin zymography was also inhibited in both cell lines (Fig. 3G). However, LAMB3 did not affect the expression or secretion of pro-MMP2. Taken together, these findings could suggest that LAMB3 induces the migration and invasion of PTC cells via EMT and MMP9 expression.

### LAMB3 regulates PI3K-mediated Akt phosphorylation

**Figure 4:**
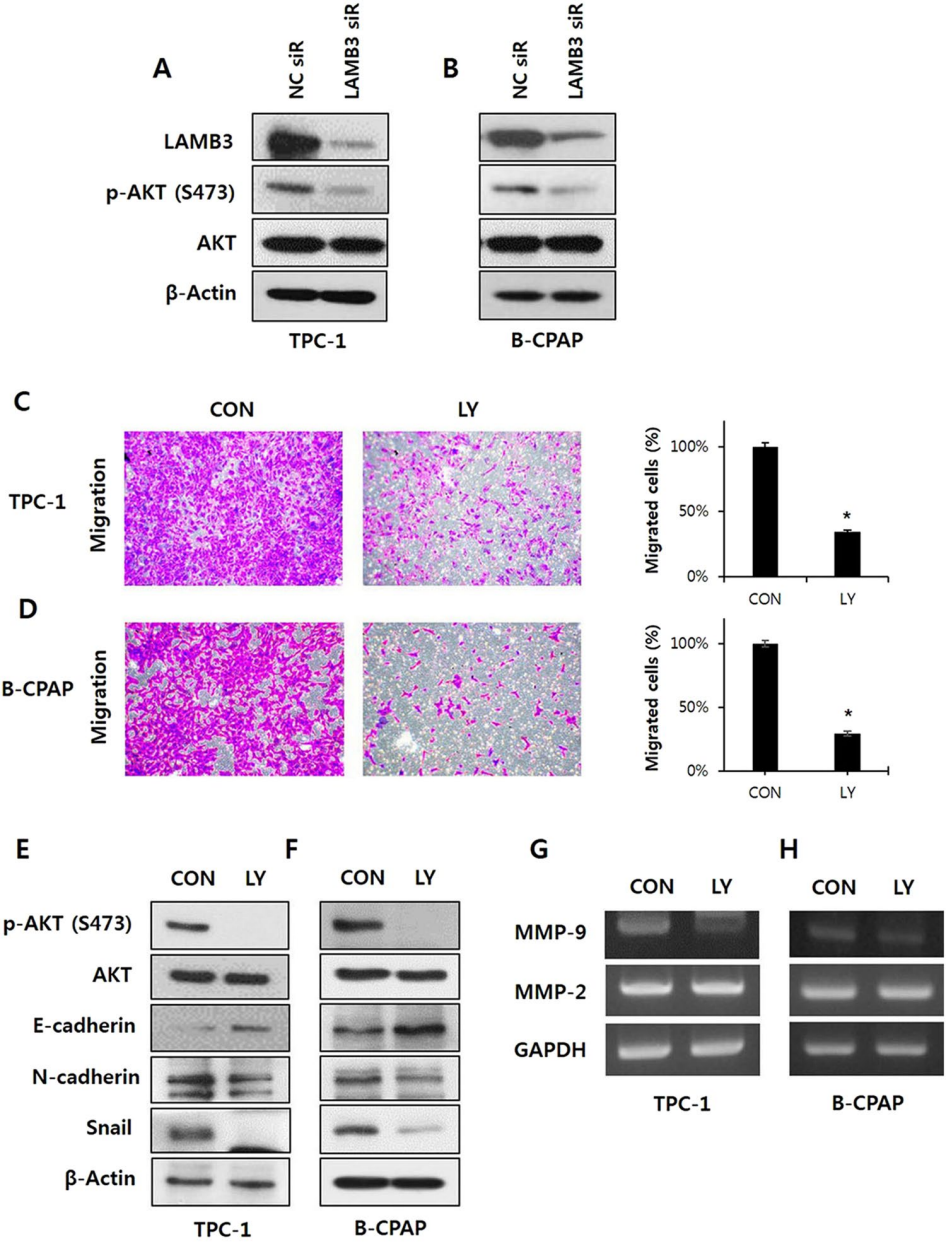
LAMB3 regulates PI3K-mediated Akt phosphorylation. Phospho-Akt and total Akt levels were examined by western blot analysis in TPC-1 (**A**) and B-CPAP cells (**B**) after LAMB3 siRNA or negative control siRNA transfection for 48 h. Migration of TPC-1 (**C**) and B-CPAP (**D**) were determined using Transwell chambers after LY294002 (15 μM) treatment for 24 h. *P < 0.05. EMT-related proteins E-cadherin, N-cadherin, vimentin, Slug, and Snail were examined by western blot analysis in TPC-1 (**E**) and B-CPAP (**F**) after LY294002 (15 μM) treatment for 24 h. E. MMP2 and MMP9 mRNA levels in TPC-1 (**G**) and B-CPAP (**H**) were analyzed by RT-PCR after LY294002 (15 μM) treatment for 24 h. Each figure is representative of three independent experiments.

We hypothesized that LAMB3 regulates cell migration and invasion via certain cellular signaling pathways. We investigated various pathways involved in tumorigenesis and/or metastasis. We observed that LAMB3 suppression significantly decreased Akt phosphorylation at serine 473, whereas the total level of Akt protein was not affected in TPC-1 and B-CPAP cells (Fig. 4A,B). PI3K/Akt signaling appears to play an important role in the progression of papillary cancers^21^. To elucidate whether Akt suppression reduces tumor cell migration in PTCs, a Transwell migration assay was performed using TPC-1 and B-CPAP cells following Akt inhibition by LY294002. Inhibition of PI3K/Akt signaling significantly suppressed TPC-1 cell migration (Fig. 4C,D). Akt inhibition by LY294002 also decreased levels of the EMT-related proteins vimentin, Slug, and Snail and increased that of the epithelial marker E-cadherin (Fig. 4E,F). The mRNA expression of MMP9 but not MMP2 was reduced by Akt inhibition (Fig. 4G,H). Collectively, these results reveal that LAMB3 promotes the migration and invasion of PTC cells via activation of the PI3K/Akt signaling pathway, which leads to EMT and MMP9 activation.

### LAMB3 leads to tumor invasion via Akt activation induced by the HGF/c-MET axis

**Figure 5:**
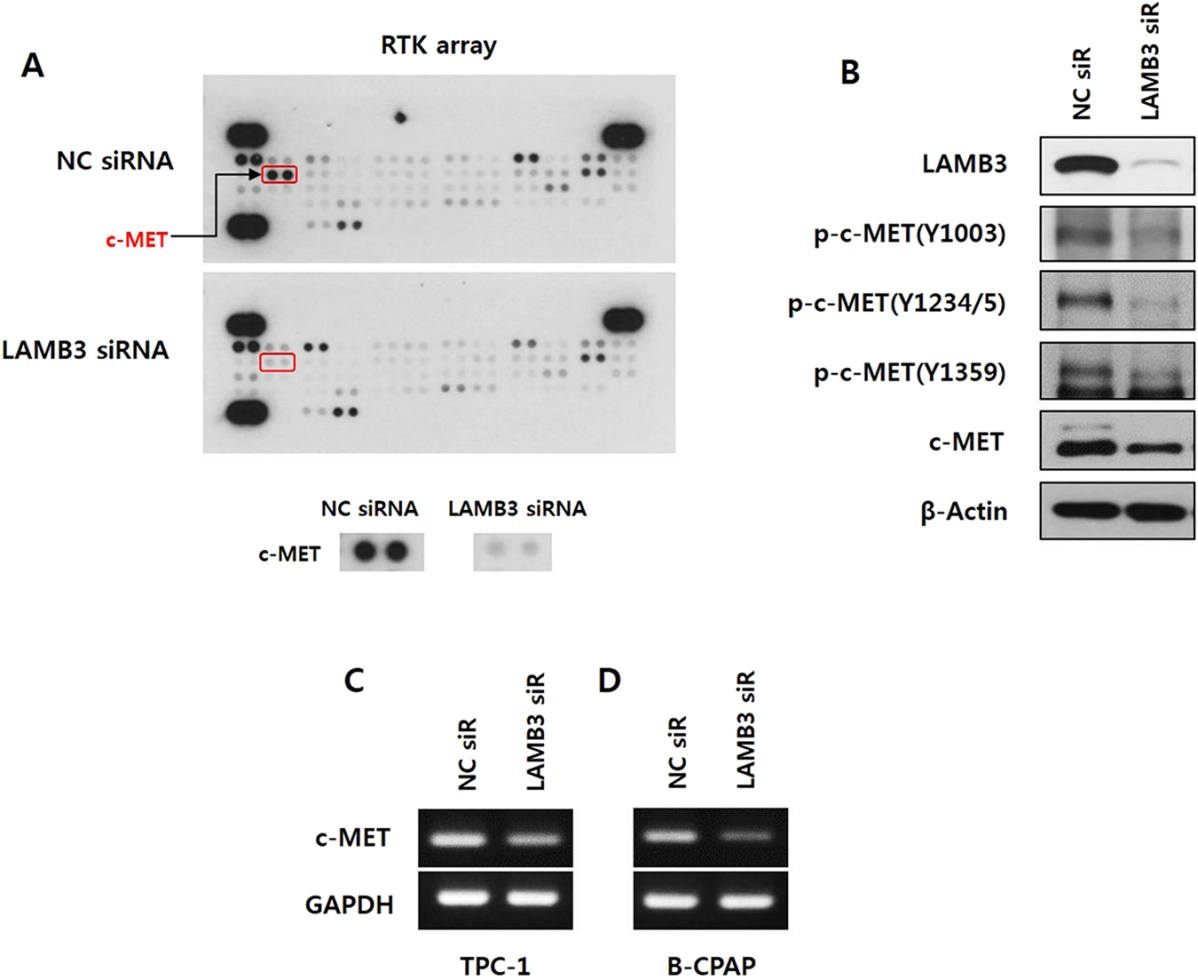
LAMB3 promotes c-MET signalling. (**A**). Lysates from TPC-1 cells transfected with LAMB3 siRNA or negative control siRNA for 48 h were analyzed by a human phospho-RTK array. (**B**). Phospho-c-MET and total c-MET were examined by western blot analysis in TPC-1 cells after LAMB3 siRNA or negative control siRNA transfection for 48 h. c-MET mRNA levels in TPC-1 (**C**) and B-CPAP (**D**) were analyzed by RT-PCR after transfection with LAMB3 siRNA or negative control siRNA. GAPDH was used as an internal control.

**Figure 6:**
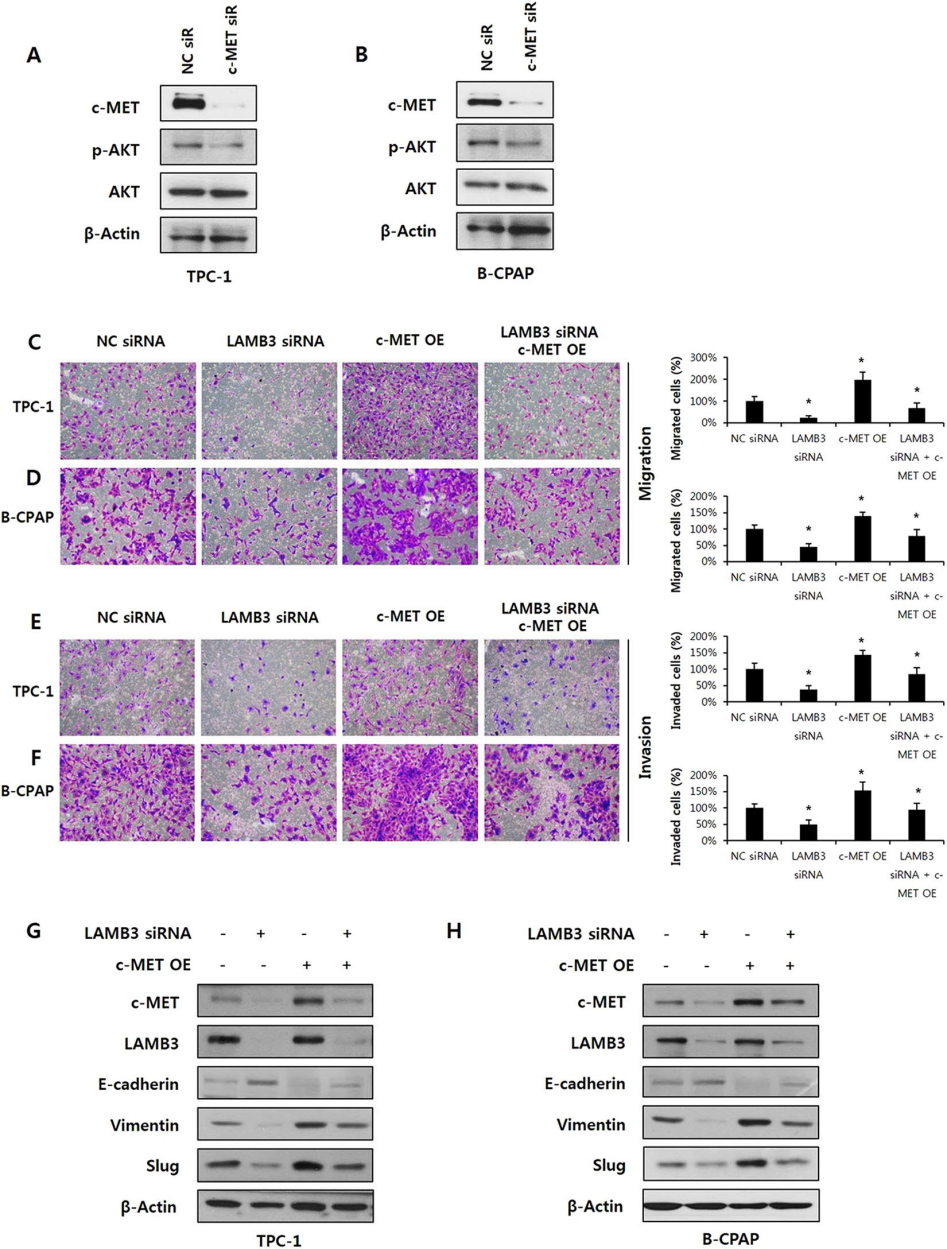
LAMB3 leads to tumor invasion via Akt activation induced by the HGF/c-MET axis. Phospho-Akt and total Akt levels were examined by western blot analysis in TPC-1 (**A**) and B-CPAP (**B**) after c-MET siRNA or negative control siRNA transfection for 48 h. TPC-1 (**C**,**E**) and B-CPAP (**D**,**F**) cells were transiently co-transfected with LAMB3 siRNA and c-MET overexpression vector or negative control for 48 h. Cells were allowed to migrate for 24 h in Transwell chambers (Cell Migration) or for 48 h in chambers coated with Matrigel (Cell Invasion). *P < 0.05. TPC-1 (**G**) and B-CPAP (**H**) cells were transiently co-transfected with LAMB3 siRNA and c-MET overexpression vector or negative control for 48 h. After transfection, the expression of EMT-related proteins including E-cadherin, Vimentin, and Slug levels, was evaluated by Western blot analysis. Each figure is representative of three independent experiments. The English in this document has been checked by at least two professional editors, both native speakers of English. For a certificate, please see: http://www.textcheck.com/certificate/xxQyRP.

To identify RTKs activated by LAMB3 in TPC-1 cells, the activation status of 49 human RTKs was analyzed using a phospho-RTK array. Among these RTKs, phospho-c-MET was markedly decreased by LAMB3 knockdown (Fig. 5A). We validated the effect of LAMB3 knockdown on c-MET activation by western blot analysis. Total cell lysates were immunoblotted with antibodies for c-MET p-Y1003, Y1234/5, Y1359, and total c-MET. LAMB3 knockdown decreased c-MET phosphorylation as well as total c-MET levels in TPC-1 cells (Fig. 5B). Interestingly, the mRNA expression of c-MET was also reduced by LAMB3 knockdown, indicating that LAMB3 regulates the transcription of c-MET in TPC-1 and B-CPAP cells (Fig. 5C,D). We investigated whether Akt was regulated by c-MET under our conditions. Phospho-Akt levels were down-regulated in cells in which c-MET had been knocked down by siRNA, suggesting that c-MET acts upstream of Akt in TPC-1 and B-CPAP cells (Fig. 6A,B). We also investigated whether over-expression of c-MET reverses the effect of LAMB3 suppression on Akt activation, EMT/MMP-9 and migration/invasion. c-Met overexpression using co-transfection of vector attenuated the effect of LAMB3 suppression on not only migration/invasion (Fig. 6C–F), but also EMT related proteins (Fig. 6G,H) in both TPC-1 and B-CPAP cells. These results demonstrate that LAMB3 activates Akt via up-regulation of c-MET, and that this LAMB3/c-MET/Akt signaling cascade induces cell invasiveness in PTCs.

## Discussion

Many studies have indicated that cell adhesion and extracellular matrix proteins contribute to the progression of cancer^22^. LM-332 (encoded by LAMA3, LAMB3, and LAMC2) has been linked to tumor invasiveness in various types of cancer^16,23–25^. In particular, LAMB3, involved in the focal adhesion pathway, is a potential biomarker of cancer invasion and metastasis^16–18^. In our data evaluating the LAMB3 expression pattern in the human thyroid cancer tissues, all the tumor tissues had higher LAMB3 expression compared to normal tissues. Given that our study had relative small number of the cases and no localization data, wider-scale studies are mandatory including immunohistochemistry to confirm the expression pattern of LAMB3 and its clinical meaning. However, our study have strength in that to our best of knowledge, we evaluated for the first time the expression of LAMB3 in the thyroid carcinoma. Next, we found that although LAMB3 expression was relatively up-regulated in three PTC cell lines (TPC-1, B-CPAP, and K1), LAMB3 knockdown by siRNA had no significant effect on the proliferation of TPC-1 or B-CPAP cells. However, the migration and invasion of PTC cells were considerably reduced by LAMB3 knockdown. These data could suggest that LAMB3 expression in PTC cells is associated with stimulation of cell migration and invasion but is not significantly involved in cell proliferation.

The PI3K/Akt pathway has a fundamental role in thyroid tumorigenesis and represents an attractive target for pharmaceutical development for a variety of malignancies^9,10,26^. LAMB3 knockdown by siRNA considerably reduced the level of phospho-Akt without altering the level of total Akt protein. EMT is a critical process in tumorigenesis, tumor invasion, and metastasis, and refers to the molecular reprogramming and phenotypic changes that characterize the transition from polarized immotile epithelial cells to motile mesenchymal cells^27^. MMPs are important mediators of cellular events, including ECM degradation and remodeling, cell proliferation, apoptosis, cell invasion/migration, and morphological changes^28^. PI3K/Akt inhibition has been shown to block sonic hedgehog pathway-induced EMT, matrix MMP9 activity, and lymphangiogenesis, reducing tumor invasiveness and metastasis^29^. The expression of a constitutive nuclear form of FOXO1, which is inhibited by Akt signaling, significantly inhibited the MMP9 activation induced by EGF^30^. In this study, we confirmed that LAMB3 regulates EMT-related proteins and MMP9 via activation of Akt signaling.

To investigate the upstream component mediating Akt regulation by LAMB3, we focused on RTKs. Aberrant expression of certain RTKs has been implicated in the development and progression of many types of cancer. These RTKs have emerged as promising drug targets for cancer therapy^31^. c-MET is an RTK that, after binding to its ligand, hepatocyte growth factor, activates a wide range of different cellular signaling pathways, including those involved in cell proliferation, motility, migration, and invasion. Activation of the HGF/c-Met axis prevents apoptosis through activation of PI3K and subsequent Akt activation^12–14^. In a previous study, we found HGF/c-Met pathway activation to be associated with subclinical central neck lymph node metastasis of PTC^15^. Using a phospho-RTK array, we found that c-MET acts upstream of Akt, indicating that LAMB3 leads to tumor invasion via Akt activation induced by the HGF/c-MET axis in PTCs.

PTCs usually possess mutations in BRAF or RET/PTC genetic rearrangements. Many of the genetic changes observed, including RET/PTC gene rearrangements and RAS and BRAF mutations, lead to activation of the MAPK and PI3K/Akt signaling pathways^8^. As shown in our results, both TPC-1 cells (carrying RET/PTC-1) and B-CPAP cells (carrying BRaf^V600E^) promoted tumor progression via LAMB3/Akt signaling, despite carrying different types of genetic alterations.

Recent reports have suggested that LAMB3 is a potential biomarker and drug target in PTC, and that monitoring several transcripts, including LAMB3, can afford high diagnostic accuracy^32,33^. In addition, our findings established that LAMB3 could promote the metastasis of PTC via activation of the PI3K/Akt pathway induced by the HGF/c-MET axis, leading to EMT and MMP9 activation. To our knowledge, this is the first report identifying a molecular mechanism of action for LAMB3 in PTC.

In conclusion, our findings could suggest that LAMB3 contributes to cancer cell migration and invasion in PTC via c-MET/Akt signaling. Elucidation of LAMB3-regulated cancer pathways may provide novel therapeutic strategies to control PTC metastasis.
